# The ability of post-chemoradiotherapy DWI ADC_mean_ and ^18^F-FDG SUV_max_ to predict treatment outcomes in head and neck cancer: impact of human papilloma virus oropharyngeal cancer status

**DOI:** 10.1007/s00432-021-03662-y

**Published:** 2021-06-22

**Authors:** S. Connor, C. Sit, M. Anjari, M. Lei, T. Guerrero-Urbano, T. Szyszko, G. Cook, P. Bassett, V. Goh

**Affiliations:** 1 School of Biomedical Engineering and Imaging Sciences, St Thomas’ Hospital, King’s College, London, SE1 7EH UK; 2 Department of Neuroradiology, Ruskin Wing, Kings College Hospital, Denmark Hill, London, SE5 9RS UK; 3 Department of Radiology, Guy’s Hospital, 2nd Floor, Tower Wing, Great Maze Pond, London, SE1 9RT UK; 4 King’s College London & Guy’s and St. Thomas’ PET Centre, London, SE1 7EH UK; 5 Department of Oncology, Guy’s Hospital, 2nd Floor, Tower Wing, Great Maze Pond, London, SE1 9RT UK

**Keywords:** Diffusion magnetic resonance imaging, Positron emission tomography and computed tomography, Head and neck neoplasms, Chemoradiotherapy, Treatment outcome

## Abstract

**Objectives:**

To evaluate the ability of post-chemo-radiotherapy (CRT) diffusion-weighted-MRI apparent diffusion coefficient (ADC_mean_) and ^18^F-FDG PET maximum standardized uptake value (SUV_max_) to predict disease-free survival (DFS) in head and neck squamous cell carcinoma (HNSCC), and to determine whether this ability is influenced by human papillomavirus oropharyngeal cancer (HPV-OPC) status.

**Methods:**

This prospective cohort observational study included 65 participants (53 male, mean ± SD age 59.9 ± 7.9 years, 46 HPV-OPC) with stage III or IV HNSCC. Primary tumour and nodal ADC_mean_ (pre-treatment, 6- and 12-weeks post-CRT) and SUV_max_ (12-weeks post-CRT) were measured. Variables were compared with 2-year DFS (independent *t*-test/Mann–Whitney test) and overall DFS (Cox regression), before and after accounting for HPV-OPC status. Variables were also compared between HPV-OPC and other HNSCC subgroups after stratifying for DFS.

**Results:**

Absolute post-CRT ADC_mean_ values predicted 2-year DFS and overall DFS for all participants (*p* = 0.03/0.03, 6-week node; *p* = 0.02/0.03 12-week primary tumour) but not in the HPV-OPC subgroup. In participants with DFS, percentage interval changes in primary tumour ADC_mean_ at 6- and 12-weeks were higher in HPV-OPC than other HNSCC (*p* = 0.01, 6 weeks; *p* = 0.005, 12 weeks). The 12-week post-CRT SUV_max_ did not predict DFS.

**Conclusion:**

Absolute post-CRT ADC_mean_ values predicted DFS in HNSCC but not in the HPV-OPC subgroup. Amongst participants with DFS, post-CRT percentage interval changes in primary tumour ADC_mean_ were significantly higher in HPV-OPC than in other HNSCC. Knowledge of HPV-OPC status is crucial to the clinical utilisation of post-CRT DWI-MRI for the prediction of outcomes.

**Supplementary Information:**

The online version contains supplementary material available at 10.1007/s00432-021-03662-y.

## Introduction

Head and neck squamous cell cancer (HNSCC) is the seventh commonest cancer (Ferlay et al. 2010). Concomitant chemo-radiotherapy (CRT) is the standard of care for the advanced disease at most head and neck tumour sites, with treatment failing at loco-regional sites in over 30% of stage III or IV tumours (Goodwin 2000).

Conventional CT and MRI evaluation is challenging in the presence of post-treatment tissue distortion (Hermans et al. 2000; Arga et al. 2006; King et al. 2013a, b). Metabolic imaging with 18F-fluorodeoxygluocose (^18^F-FDG) PET-CT (Sheikhbahaei et al. 2015) may overcome these limitations and is widely used to achieve earlier detection of residual disease. Quantitative post-treatment ^18^F-FDG PET-CT SUV_max_ (Moeller et al. 2009; Chan et al. 2012; Sherriff et al. 2012; Castelli et al. 2016; Kim et al. 2016; Matoba et al. 2017) has been shown to predict treatment failure and survival outcomes.

Quantitative diffusion-weighted MRI (DW-MRI) may provide alternative post-CRT imaging variables for the prediction of treatment success. Cellular tumour impedes diffusion of water molecules, resulting in lower ADC values (Chawla et al. 2009), and it has been hypothesised that a reduction in cellularity and progressive necrosis with successful treatment leads to a greater rise in ADC values. A number of studies have evaluated post-treatment tumour ADC_mean_ as a biomarker (Kim et al. 2009; King et al. 2010; Vandecaveye et al. 2012; Schouten et al. 2014; Marzi et al. 2017; Brenet et al 2020) with increased absolute ADC_mean_, or a greater percentage interval increase in ADC_mean_ from pre-treatment values, being associated with the disease control (King et al. 2010; Vandecaveye et al. 2012; Brenet et al 2020). Since DW-MRI probes a different biological process to ^18^F-FDG PET-CT, the two modalities may be complementary in stratifying the risk of residual or recurrent disease (Preda et al. 2016).

Human papillomavirus oropharyngeal cancer (HPV OPC) status is a potential confounding factor in these studies. HPV OPC has unique histopathological characteristics (Chernock et al. 2009) and differing tumour metabolism (Krupar 2014) which influence ADC measures (Chan et al. 2016; Driessen et al. 2016) and post-treatment SUV_max_ (Moeller et al. 2009; Zhang et al. 2010; Castelli et al. 2016; Vainshtein et al. 2014; Helsen et al. 2018), whilst resulting in improved clinical outcomes (Chaturvedi et al. 2011). However, the HPV-OPC status is rarely documented in studies of post-treatment quantitative DW-MRI (Marzi et al. 2017) or post-treatment ^18^F-FDG PET-CT in predicting HNSCC outcomes (Sherriff et al. 2012; Kim et al. 2016; Matoba et al. 2017). HPV OPC has not been previously considered as a co-variant in the studies of post-treatment ADC values and their prognostic significance in HNSCC.

In this study, we first aimed to determine whether 6- and 12-week post-CRT ADC_mean_ values (absolute values and percentage interval increase in values) and 12-week post-CRT SUV_max_ were able to predict DFS in stage III/IV HNSCC. Second, we explored whether this prediction was influenced by HPV OPC status, and whether these quantitative post-CRT DW-MRI variables and ^18^F-FDG PET-CT differed between HPV-OPC and other HNSCC, after stratifying for disease-free survival (DFS) status.

## Methods

### Participants

Participants were recruited to a prospective single-centre cohort observational study between May 2014 and July 2017 (http://www.controlled-trials.com/ISRCTN58327080). Research Ethics Committee approval (REC reference 13/LO/1876) and informed consent was obtained.

Participants were eligible if: (1) there was a histologically confirmed stage III or IV primary HNSCC without distant metastatic disease (2) a 1cm^2^ area of measurable primary tumour and/or nodal tumour on the basis of standard clinico-radiological staging, and (3) curative CRT was planned. Exclusion criteria included prior CRT, Eastern Cooperative Oncology Group (ECOG) performance status > 2, inability to provide informed consent, known allergy to gadolinium-based contrast medium and eGFR < 30 ml/min.

Sample size was calculated to demonstrate a difference in the percentage change in ADC values between participants with and without DFS at 2 years. It was assumed that 70% of the participants would be disease-free at 2 years (Goodwin 2000) with the standard deviation of the percentage change in ADC values being 20% (Kim et al. 2009; King et al. 2010; Marzi et al. 2017; Schouten et al. 2014; Vandecaveye et al. 2012). A sample size of 70 was projected to show a 15% difference between those with and without 2-year DFS assuming 5% significance level and 80% power.

### HPV status, biopsies and treatment

HPV status was analysed for all OPC as per standard of care. Non-OPC HNSCC was not routinely tested for HPV status, according to international guidelines (Fakhry et al. 2018). HPV status was evaluated with p16 testing using an immune-stain or high-risk HPV DNA testing using in situ hybridisation. HPV status was analysed for 49/49 oropharyngeal and 2/16 other HNSCC. Diagnostic biopsies were obtained from the primary tumour (*n* = 56), lymph node (*n* = 7) or both (*n* = 2).

### Imaging

Patients underwent (1) MRI before treatment and at 6- and 12-weeks post-CRT as per the study protocol and (2) ^18^F-FDG PET-CT imaging at 12-weeks post-CRT as per institutional practice.

#### MRI: protocol and technique

Patients underwent standard institutional head and neck soft tissue protocol MRI on a 1.5 T scanner (Siemens Magnetom Aera) using a surface phased array neck coil. An additional research echo-planar diffusion-weighted sequence was acquired in the axial plane with the following *b*-values: 0, 50, 100, 800 and 1500 s/mm^2^ (supplementary table).

#### MRI: processing and analysis

**Fig. 1:**
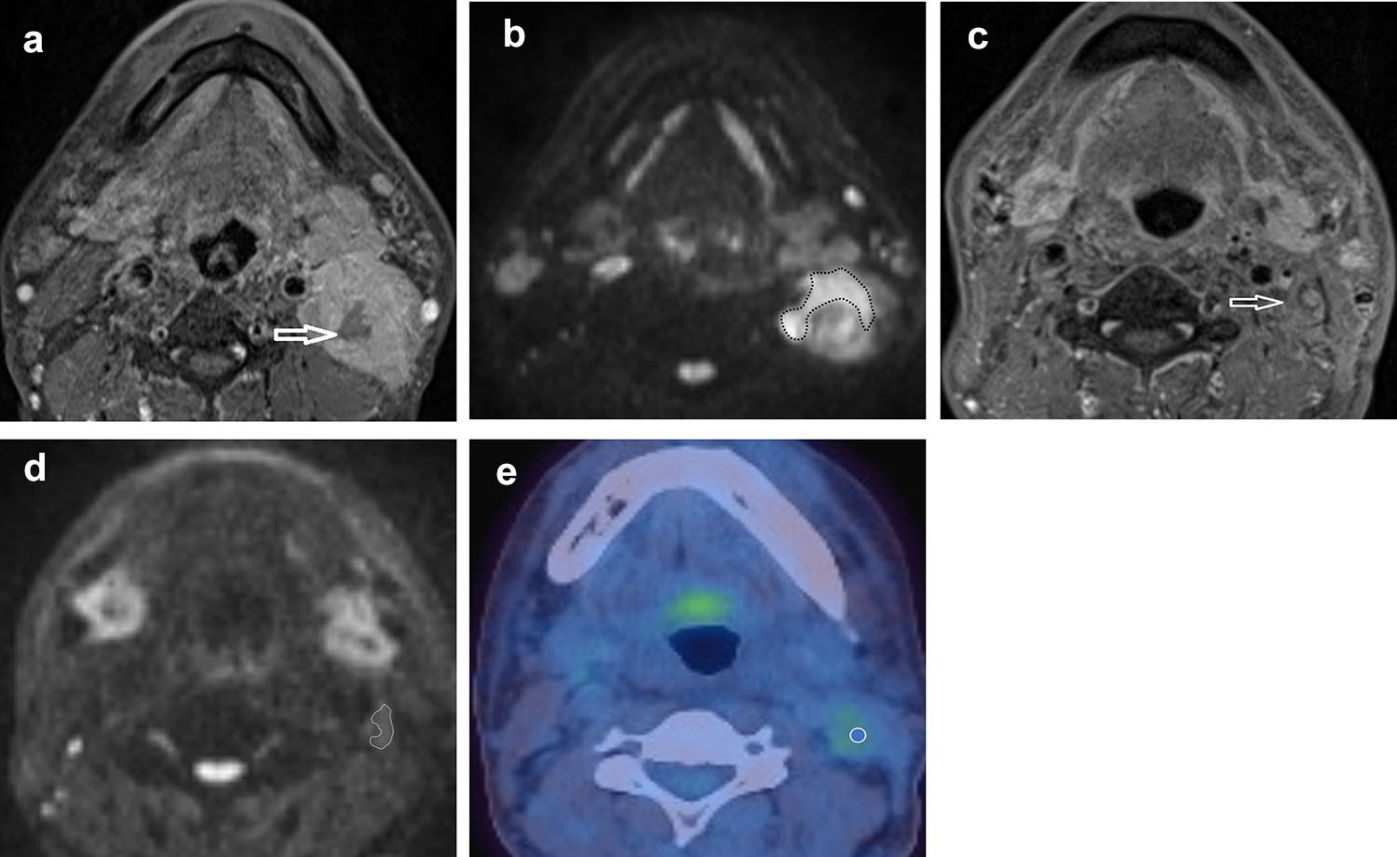
A HPV negative participant with a partially necrotic left level 2 lymph node. **a** T1w post gadolinium axial image pre-treatment demonstrates the lymph node (arrow). **b** *b* = 800 s/mm^2^ map from DW-MRI pre-treatment indicating the lymph node ROI as the increased DWI signal whilst avoiding the necrotic area. **c** T1w post gadolinium axial image at 12 weeks post-treatment demonstrates the lymph node to be of reduced size (arrow). **b** *b* = 800 s/mm^2^ map from DW-MRI at 12 weeks post-treatment indicating the lymph node ROI as the increased DWI signal. **e** ^18^F-FDG PET-CT study at 12 weeks post-treatment demonstrating the 6 mm VOI at the site of mild ^18^F-FDG uptake in the lymph node

**Fig. 2:**
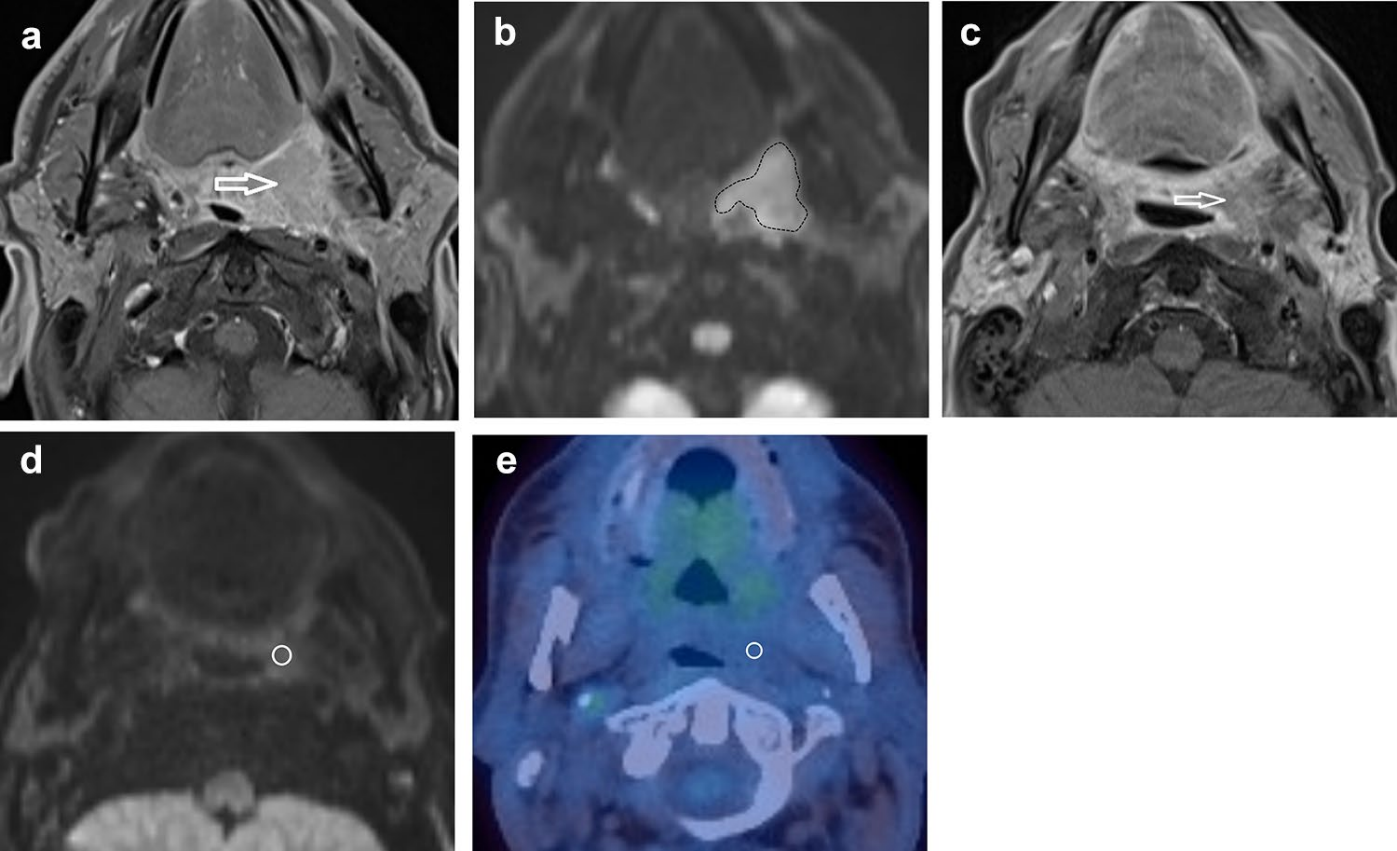
A HPV-positive participant with a left palatine tonsillar tumour. **A** T1w post gadolinium axial image pre-treatment demonstrates the left palatine tonsillar tumour (arrow). **b** *b* = 800 s/mm^2^ map from DW-MRI pre-treatment indicating the primary tumour ROI as the increased DWI signal. **C** T1w post gadolinium axial image at 12 weeks post-treatment demonstrates the primary tumour to be of reduced size (arrow). **b** *b* = 800 s/mm^2^ map from DW-MRI at 12 weeks post-treatment indicating the primary tumour standardised 6 mm ROI since there is no increased DWI signal relative to adjacent oropharyngeal tissue. **e** ^18^F-FDG PET-CT study at 12 weeks post-treatment demonstrating the 6 mm VOI at the primary tumour. Since there is no ^18^F-FDG uptake to target, it is placed with guidance from the MRI study

The ROIs were placed by a radiologist (3 years’ experience) under the supervision of another radiologist (21 years’ experience). The first 24 participants were also independently analysed by a further radiologist (5 years’ experience) to assess for inter-observer agreement. ROIs were placed individually within the primary tumour and/or the largest pathological lymph node (Figs. 1, 2) using OsiriX v8.0.2, open-source Mac-based medical image processing software. ROIs were placed on the pre-treatment, 6-week (ADC_mean_6) and 12-week (ADC_mean_12) post-treatment MRI studies using the DWI *b* = 800 s/mm^2^ map, but with access to other MRI sequences. When a focus of increased DWI signal was not evident on post-treatment images, a standardised 6 mm diameter ROI was placed at its original location. An ADC map was generated from the *b* 100 and *b* 800 s/mm^2^ images. A ROI was also placed within the cervical spinal cord on the ADC map as a reference.

#### ^18^F-FDG PET-CT: protocol and technique

^18^F-FDG PET-CT was performed as per standard clinical practice. Patients were fasted for at least 6 h prior to administration of 350–400 MBq ^18^F-FDG. PET/CT scans were acquired 90 min after injection from the upper thigh to the base of the skull with additional local views of the head and neck performed on one of two PET-CT scanners (Siemens mCT Flow VST or GE Discovery DST 710) (supplementary table).

#### ^18^F-FDG PET/CT: processing and analysis

A 6 mm diameter volume of interest (VOI) was placed by a radiologist (3 years’ experience), under the supervision of another radiologist (16 years’ experience). VOIs were placed at the site of most intense FDG uptake within either the primary lesion and/or the largest lymph node, which were matched to the ROI placed for the MRI analysis (Figs. 1, 2). If there was reduced uptake on the post-treatment images relative to background, a 6 mm VOI was placed at the same site as the post-treatment MRI ROI. If necrosis was identified within a lesion, the area of necrosis was excluded. The SUV_max_ was calculated with semi-automated software on a Hermes workstation (Hermes Gold 3, Stockholm).

### Treatment and treatment outcome

Intensity-modulated radiotherapy (IMRT) was delivered as 7-Gy in 35 fractions (2 Gy per fraction delivered once daily, 5 days a week). Concomitant intravenous cisplatin at a dose of 35 mg/m2 every 7 days, starting on day 1 of radiotherapy, was used for all patients with adequate GFR and no contraindications to cisplatin (*n* = 47) with carboplatin being used if measured GFR < 50 or if a patient had a history of hearing impairment (*n* = 16). Two patients received radiotherapy alone. The time from the completion of treatment to disease progression was recorded for those participants without DFS and time from the completion of treatment to the latest follow-up was recorded for those with DFS. The 2-year DFS was recorded for all participants. A 12-week PET-CT study was standard of care with clinical assessment at 1- and 2-years post-CRT. Treatment failure was determined by cytological or histological confirmation or serial progression on imaging.

### Statistical analysis

Analysis was performed using Stata (version 15.1) with a *p* value of < 0.05 being considered statistically significant.

The percentage interval changes in ADC_mean_ (%ADC_mean_0–6, %ADC_mean_0–12,%ADC_mean_6–12, respectively), were calculated.

The %ADC_mean_0–6, %ADC_mean_0–12, %ADC_mean_6–12 ADC_mean_6, ADC_mean_12 and SUV_max_12, at primary tumour and nodal locations, were compared with survival outcomes using two different methods. First, they were compared between participants with and without 2-year DFS using the independent t-test, if variables were normally distributed, and the Mann–Whitney test if they were not normally distributed. Second, the association between the imaging variables and DFS outcome was evaluated using Cox regression analysis after censoring patients without DFS at the time of the last follow-up. These comparisons with DFS were performed for all participants and subsequently for the HPV OPC and other HNSCC subgroups alone.

No multiple testing correction for these pre-designed “planned comparisons” was deemed appropriate in this exploratory study.

Receiver operating characteristic (ROC) analysis was used to identify the area under the curve (AUC), optimal threshold and sensitivity/specificity/ positive predictive value (PPV)/negative predictive value (NPV) for any parameters predictive of 2-year DFS. The optimal threshold was chosen as the point which maximised the combination of sensitivity and specificity. Hazard ratios were also calculated for variables predictive of overall DFS from the Cox regression analysis. These represent the relative chance in the hazard (risk) of disease progression at any time for a specified increase in the given variable.

The variables in HPV OPC and other HNSCC subgroups were compared with each other after stratifying for presence or absence of DFS. Continuous variables were compared using the independent t-test if normally distributed, and the Mann–Whitney test if not normally distributed.

The primary tumour and lymph node %ADC_mean_0–12, %ADC_mean_6–12 and ADC_mean_12 were correlated with SUV_max_ 12 using Pearsons correlation coefficient.

The Intra-class correlation coefficients (ICCs) were evaluated for interobserver agreement.

## Results

### Participants and descriptive statistics

**Fig. 3:**
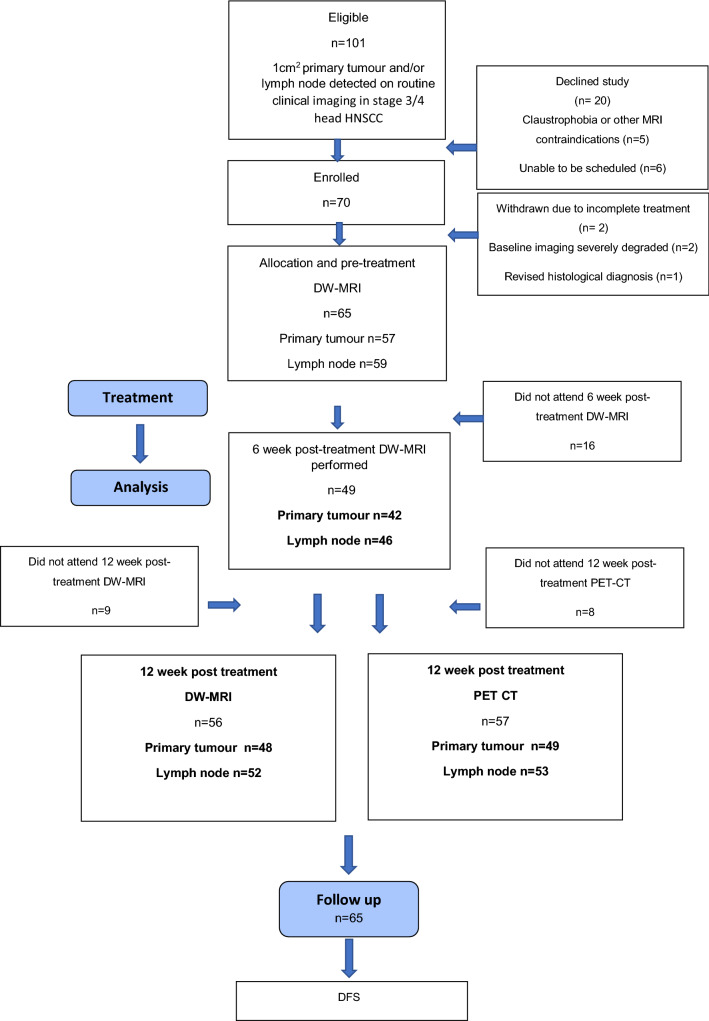
Participant consort flow diagram

The participant consort flow diagram is demonstrated in Fig. 3.

There were 70 subjects enrolled out of 101 eligible, but five were subsequently withdrawn. Of the 65 participants (53 male, 12 female, mean age 59.9 ± 7.9 years), there were 11 with stage III disease (17%) and 54 with stage IV (83%). Participant characteristics including primary site, nodal staging and HPV status are summarised in Table 1. There were 46/65 patients with HPV-OPC.

**Table 1:** Site, subsite, TN stage and HPV status for the 65 participants

	Subsite			T stage					N stage					HPV status		
				T0	T1	T2	T3	T4	NO	N1	N2A	N2B	N2C	+ ve	−ve	No test
Oro pharynx (n = 49)	Tongue base	Tonsil	Soft palate													
	29	19	1	1	7	18	7	16	3	4	3	29	10	46	3	0
Larynx (*n* = 10)	Supra glottic	Trans glottic														
	7	3					8	2	0	5	3	1	1	0	2	8
Hypopharynx (*n* = 6)	Piriform fossa															
	6					3	2	1				4	2	0	0	6

The number of primary tumours and lymph nodes analysed, the ICCs for the sample of ROIs performed by two observers, and the cervical cord ROI ADC_mean_ values at the different DW-MRI time points are indicated in Table 2.

**Table 2:** Number of participants, number of primary tumours and lymph nodes analysed, inter-observer agreement, and cervical cord ROI ADC_mean_ values at the different DW-MRI time points

	Pre-treatment	6 weeks	12 weeks
Participants (*n*)	65	49	56
Primary tumour/lymph node/both (*n*)	6/8/51	3/7/39	4/8/44
Primary tumour/lymph node ICC ADC_mean_	0.97/0.98	0.98/0.98	0.98/0.94
Cervical cord ROI ADC_mean_ ± SD (× 10^–6^ mm^2^/s)	1004 ± 79	1009 ± 29.2	994 ± 28.7

The median follow-up was 4.1 [3.05, 5.0] years post treatment. Ten participants had progressive disease within two years of completing CRT (isolated nodal recurrence (*n* = 4); nodal, primary and distal metastatic recurrence (*n* = 1); isolated primary recurrence (*n* = 1) and distal metastatic recurrence (*n* = 4). The median time to recurrence was 0.51 [0.30, 0.72] years post-treatment. There were no other cases of progressive disease within the duration of the study follow-up.

### Comparison of post-CRT ADC_mean_ variables and SUV_max_ with 2-year and overall DFS

**Fig. 4:**
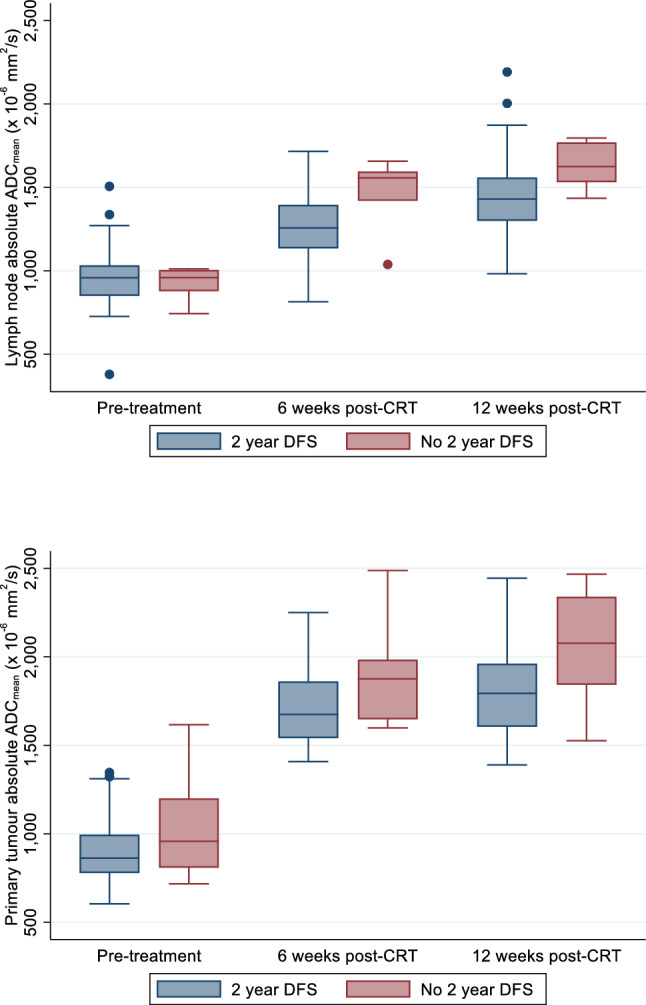
Box plot illustrating the lymph node and primary tumour absolute ADC_mean_ values at pre-treatment, 6 weeks post-CRT and 12 weeks post-CRT in participants with and without 2 year DFS

Table 3 demonstrates the comparison of post-CRT percentage interval changes in ADC_mean_, absolute ADC_mean_ values and SUV_max_, with DFS outcomes for all participants, the HPV-OPC subgroup and the other HNSCC subgroup. A box plot (Fig. 4) illustrates the lymph node and primary tumour absolute ADC_mean_ values at pre-treatment, 6 weeks post-CRT and 12 weeks post-CRT in participants with and without 2 year DFS.

**Table 3:** Comparison of post CRT ^18^F-FDG PET-CT (SUV_max_ 12) and DW-MRI parameters (%ADC_mean_ 0–6, %ADC_mean_ 0–12, ADC_mean_ 6 and ADC_mean_ 12) between participants with and without 2-year and overall DFS: all participants, HPV OPC and other HNSCC

Variable	No 2 year DFS		2 year DFS		*p*-value 2 year v no 2 year DFS^a^	*p*-value overall DFS^b^
	*n*	Summary	*n*	Summary		
**All participants**						
Interval change %ADC_mean_						
Lymph node %ADC_mean_ 0–6	6	62 [41, 81]	40	37 [10, 55]	0.12	0.15
Lymph node %ADC_mean_ 0–12	6	77 [57, 82]	46	53 [25, 77]	0.10	0.53
Lymph node %ADC_mean_ 6–12	5	6 [0, 13]	37	12 [2, 27]	0.36	0.43
Primary tumour %ADC_mean_ 0–6	7	100 ± 67	35	98 ± 43	0.91	0.91
Primary tumour %ADC_mean_ 0–12	6	112 ± 58	42	109 ± 44	0.88	0.89
Primary tumour %ADC_mean_ 6–12	5	10 ± 17	32	5 ± 11	0.39	0.35
Absolute ADC_mean_ (× 10^–6^ mm^2^/s)						
Lymph node ADC_mean_ 0	8	930 ± 94	51	955 ± 182	0.70	0.65
Lymph node ADC_mean_ 6	6	1471 ± 226	40	1268 ± 194	**0.02**	**0.03**
Lymph node ADC_mean_ 12	6	1630 ± 143	46	1441 ± 254	0.08	0.09
Primary tumour ADC_mean_ 0	10	958 [808, 1201]	47	863 [779, 996]	0.16	0.19
Primary tumour ADC_mean_ 6	7	1897 ± 303	36	1719 ± 222	0.07	0.07
Primary tumour ADC_mean_ 12	6	2055 ± 365	42	1798 ± 243	**0.03**	**0.03**
SUV_max_						
Lymph node SUV_max_ 12	6	2.1 ± 0.5	47	1.9 ± 0.6	0.48	0.51
Primary tumour SUV_max_ 12	6	3.4 ± 1.2	43	2.9 ± 0.8	0.21	0.19
**HPV OPC**						
Interval change %ADC_mean_						
Lymph node %ADC_mean_ 0–6	3	81 [3, 115]	33	37 [16, 54]	0.32	0.14
Lymph node %ADC_mean_ 0–12	3	82 [52, 127]	37	60 [27, 79]	0.21	0.18
Lymph node %ADC_mean_ 6–12	2	3 [0, 6]	31	10 [2, 28]	0.26	0.34
Primary tumour %ADC_mean_ 0–6	3	141 ± 83	28	107 ± 41	0.23	0.19
Primary tumour %ADC_mean_ 0–12	3	148 ± 60	32	119 ± 41	0.41	0.29
Primary tumour %ADC_mean_ 6–12	2	2 ± 14	26	5 ± 11	0.70	0.68
Absolute ADC_mean_ (× 10^–6^ mm^2^/s)						
Lymph node ADC_mean_ 6	3	1496 ± 311	33	1281 ± 192	0.35	0.34
Lymph node ADC_mean_ 12	3	1562 ± 128	37	1444 ± 257	0.44	0.45
Primary tumour ADC_mean_ 6	3	1911 ± 500	29	1710 ± 225	0.20	0.15
Primary tumour ADC_mean_ 12	3	1885 ± 382	32	1801 ± 258	0.61	0.61
SUV_max_						
Lymph node SUV_max_ 12	3	1.9 ± 0.5	37	1.9 ± 0.5	0.98	0.96
Primary tumour SUV_max_ 12	3	3.4 ± 0.6	32	3.0 ± 0.8	0.42	0.41
**Other HNSCC**						
Interval change %ADC_mean_						
Lymph node %ADC_mean_ 0–6	3	54 [41, 70]	7	31 [− 8, 67]	0.21	0.67
Lymph node %ADC_mean_ 0–12	3	75 [57, 79]	9	40 [24, 47]	0.08	0.92
Lymph node %ADC_mean_ 6–12	3	13 [-8, 26]	6	13 [-15, 27]	1.00	0.75
Primary tumour %ADC_mean_ 0–6	4	70 ± 39	7	63 ± 31	0.74	0.70
Primary tumour %ADC_mean_ 0–12	3	76 ± 30	10	76 ± 36	0.99	0.92
Primary tumour %ADC_mean_ 6–12	3	15 ± 18	6	2 ± 13	0.27	0.20
Absolute ADC_mean_ (× 10^–6^ mm^2^/s)						
Lymph node ADC_mean_ 6	3	1545 ± 119	7	1205 ± 207	**0.03**	0.11
Lymph node ADC_mean_ 12	3	1699 ± 145	9	1428 ± 256	0.12	0.12
Primary tumour ADC_mean_ 6	4	1886 ± 128	7	1753 ± 220	0.31	0.36
Primary tumour ADC_mean_ 12	3	2225 ± 316	10	1787 ± 201	**0.01**	**0.04**
SUV_max_						
Lymph node SUV_max_ 12	3	2.2 ± 0.6	10	1.7 ± 0.5	0.22	0.21
Primary tumour SUV_max_ 12	3	3.5 ± 1.8	11	2.8 ± 0.9	0.34	0.30

Significance for the bold values *p*<0.05

Summary statistics are: number (percentage), mean ± standard deviation or median [inter-quartile range]

^a^Continuous variables were compared using the independent *t*-test if normally distributed, and the Mann–Whitney test if not normally distributed

^b^Cox regression analysis

The lymph node absolute ADC_mean_ at 6 weeks (*p* = 0.02) and primary tumour absolute ADC_mean_ at 12 weeks (*p* = 0.03) was predictive of 2-year DFS for all participants with higher values of being associated with an increased risk of 2-year DFS. The lymph node absolute ADC_mean_ at 6 weeks predicted 2-year DFS with AUC of 0.77 with an optimum threshold of 1405 10^–6^ mm^2^/s and sensitivity/specificity/PPV/NPV of 83%/80%/39%/97%, respectively. The primary tumour absolute ADC_mean_ at 12 weeks predicted 2-year DFS with AUC of 0.70 with an optimum threshold of 1840 10^–6^ mm^2^/s and sensitivity/specificity/PPV/NPV of 83%/57%/22%/96%, respectively. Application of these thresholds predicted 5 of the 6 patients with disease progression at 2 years.

**Fig. 5:**
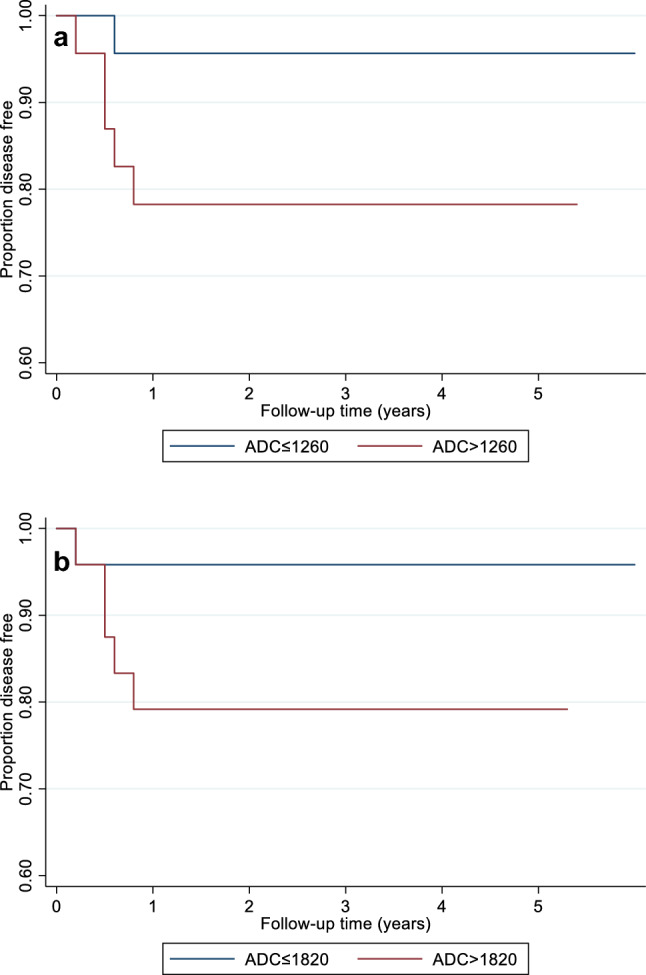
Kaplan–Meier plots illustrate the impact of lymph node absolute ADC_mean_ at 6 weeks (**a**) and primary tumour absolute ADC_mean_ at 12 weeks (**b**) on DFS. For the purposes of illustration of the results, the patients were split into two equal-sized groups by the median ADC value for each parameter

The lymph node absolute ADC_mean_ at 6 weeks (*p* = 0.03) and primary tumour absolute ADC_mean_ at 12 weeks (*p* = 0.03) were also predictive of overall DFS for all participants according to Cox regression analysis. A 100 × 10^–6^ mm^2^/s higher lymph node ADC_mean_ at 6 weeks was associated with the risk of DFS increasing by 61% (4–149%; 95% CI), whilst a 100 × 10^–6^ mm^2^/s higher primary tumour absolute ADC_mean_ at 12 weeks was associated with the risk of DFS increasing by a 38% (3–184%; 95% CI) at any time. Kaplan–Meier plots illustrate the impact of lymph node absolute ADC_mean_ at 6 weeks and primary tumour absolute ADC_mean_ at 12 weeks on the DFS (Fig. 5).

None of the percentage interval changes in ADC_mean_ or absolute ADC_mean_ values variables were able to predict DFS in the HPV OPC subgroup (Table 3).

The lymph node absolute ADC_mean_ at 6 weeks was associated with 2-year DFS (*p* = 0.03) and the primary tumour absolute ADC_mean_ at 12 weeks was significantly associated with both 2-year DFS and DFS (*p* = 0.01; *p* = 0.04) in the other HNSCC subgroup. However, there should be some caution exercised in interpreting these results due to the small number of participants in this subgroup (Table 3).

The 12-week post-CRT SUV_max_ did not predict DFS for either HPV-OPC or other HNSCC.

### Comparison of post-CRT ADC_mean_ variables and SUV_max_ between HPV-OPC and other HNSCC when stratified by  DFS

Table 4 demonstrates the comparison of post-CRT percentage interval changes in ADC_mean_, absolute ADC_mean_ values and SUV_max_, between HPV-OPC and other HNSCC, in participants with DFS.

**Table 4:** Comparison of post CRT ^18^F-FDG PET-CT (SUV_max_ 12) and DW-MRI parameters (%ADC_mean_ 0–6, %ADC_mean_ 0–12, ADC_mean_ 6 and ADC_mean_ 12) between HPV OPC and other HNSCC participants with DFS

Variable	Other HNSCC		HPV OPC		*p*-value
	*n*	Summary	*n*	Summary	
DFS					
** Interval change %ADC**_**mean**_					
Lymph node %ADC_mean_ 0–6	7	31 [− 8, 67]	33	37 [16, 55]	0.61
Lymph node %ADC_mean_ 0–12	9	40 [24, 47]	37	60 [27, 79]	0.30
Lymph node %ADC_mean_ 6–12	6	13 [− 15, 27]	31	10 [2, 28]	0.74
Primary tumour %ADC_mean_ 0–6	7	63 ± 31	28	107 ± 41	**0.01**
Primary tumour %ADC_mean_ 0–12	10	76 ± 36	32	119 ± 41	**0.005**
Primary tumour %ADC_mean_ 6–12	6	2 ± 13	26	5 ± 11	0.58
**Absolute ADC**_**mean**_ **(× 10**^**–6**^ **mm**^**2**^**/s)**					
Lymph node ADC_mean_ 6	7	1205 ± 207	33	1281 ± 192	0.35
Lymph node ADC_mean_ 12	9	1428 ± 256	37	1444 ± 257	0.86
Primary tumour ADC_mean_ 6	7	1754 ± 220	29	1710 ± 225	0.65
Primary tumour ADC_mean_ 12	10	1787 ± 201	32	1801 ± 258	0.87
**SUV**_**max**_					
Lymph node SUV_max_ 12	10	1.7 ± 0.5	37	1.9 ± 0.5	0.22
Primary tumour SUV_max_ 12	11	2.8 ± 0.9	32	3.0 ± 0.8	0.46

Significance for the bold values *p* < 0.05

Summary statistics are: number (percentage), mean ± standard deviation or median [inter-quartile range]

In participants with DFS, the percentage interval changes in ADC values at the primary tumour site were significantly higher in HPV OPC than in other HNSCC, both at 6 weeks (%ADC_mean_0–6; *p* = 0.01) and at 12 weeks (%ADC_mean_0–12; *p* = 0.005).

There was no significant difference between HPV OPV and other HNSCC subgroups for the primary tumour absolute ADC_mean_, any of the lymph node ADC_mean_ variables or the 12-week SUV_max_ in participants with 2-year DFS.

Due to the small sample of participants without DFS, a formal statistical comparison was not performed between HPV OPV and other HNSCC variables, although primary tumour percentage interval changes in ADC_mean_ values were again noted to be higher in HPV OPC. For instance, at 12 weeks, the percentage interval change in ADC_mean_ for HPV-OPC (141 ± 83% × 10^–6^ mm^2^/s; *n* = 3) was higher than that for other HNSCC (70 ± 39% × 10^–6^ mm^2^/s; *n* = 4).

### Correlation between 12 week ADC_mean_ variables and 12 week SUV_max_

The correlation between %ADC_mean_0–12, %ADC_mean_6–12 and ADC_mean_12 and SUV_max_12 at the primary tumour and lymph node sites is shown in Table 5. There was no significant correlation between SUV_max_12 and ADC_mean_12 or its interval change with *p* = 0.50–0.82 at the lymph node site and *p* = 0.52–0.71 at the tumour site.

**Table 5:** Correlation between 12 week SUV_max_ and 12 week ADC_mean_ parameters

12 week ADC_mean_ parameter	*n*	Pearson’s correlation coefficient	*p*-value
Lymph node ADC_mean_ 12	52	– 0.09	0.53
Lymph node %ADC_mean_ 0–12	52	– 0.10	0.50
Lymph node %ADC_mean_ 6–12	42	– 0.04	0.82
Primary tumour ADC_mean_ 12	48	– 0.06	0.71
Primary tumour %ADC_mean_ 0–12	48	– 0.07	0.63
Primary tumour %ADC_mean_ 6–12	37	– 0.11	0.52

## Discussion

The absolute 6 week lymph node and 12 week primary tumour ADC_mean_ values were able to predict 2-year DFS and overall DFS for the whole cohort, but not for the HPV-OPC subgroup. The percentage changes in primary tumour ADC_mean_ from pre-treatment to 6- and 12-week post-CRT were unable to predict DFS, and were significantly higher in successfully treated HPV-OPC primary tumours compared to successfully treated HNSCC at other sites. The 12-week post-CRT SUV_max_ did not predict DFS overall or for either subgroup and was not influenced by HPV-OPC status.

Almost 90% of HNSCC recurrences following CRT develop within 2 years (Chang et al. 2017). Timely intervention is required in order that progressive loco-regional disease can be cured with salvage surgery. Metabolic imaging with ^18^F-FDG PET-CT has evolved as a tool for the post-treatment evaluation of HNSCC but is generally delayed for at least 12 weeks due to the potential for false-positive resulting from early post-treatment inflammatory changes (Mehanna et al. 2016). Although qualitative interpretative criteria are most frequently applied (Koksel et al. 2019; Krabbe et al. 2009; Marcus et al. 2014; Porceddu et al. 2011; Sjövall et al. 2016; Zhong et al. 2020), quantitative analysis of SUV_max_ with ^18^F-FDG PET-CT has been shown to have prognostic significance (Moeller et al. 2009; Chan et al. 2012; Sherriff et al. 2012; Castelli et al. 2016; Kim et al. 2016; Matoba et al. 2017) and multi-objective radiomics models have also been applied in this setting (Zhang et al.^2018^). However, we were unable to demonstrate the value of 12-week post-CRT SUV_max_ in predicting DFS in our predominantly HPV-OPC cohort.

Quantitative DW-MRI has been proposed as an alternative prognostic biomarker for the assessment of early HNSCC treatment response to CRT. ADC_mean_ values or their interval changes from pre-treatment baseline studies have been evaluated from both intra-treatment (Kim et al. 2009; King et al. 2010; Berrak et al. 2011; Vandecaveye et al. 2012; King et al. 2013a, b; Matoba et al. 2014; Schouten et al. 2014; Ding et al. 2015; Galbán et al. 2015; Wong et al. 2016; Marzi et al. 2017; Paudyal et al. 2017) and post-treatment (King et al. 2010; Vandecaveye et al. 2012; Kim et al. 2014; Schouten et al. 2014; Marzi et al. 2017; Brenet et al 2020) DW-MRI studies, in an attempt to predict CRT outcomes. The majority of studies have found that increased absolute ADC_mean_ values or a greater rise in ADC_mean_ from pre-treatment to either intra-treatment values (Kim et al. 2009; Berrak et al. 2011; King et al. 2013a, b; Matoba et al. 2014; Ding et al. 2015; Marzi et al. 2017; Cao et al. 2019) are predictive of treatment success, however, this is not a universal finding (Galbán et al. 2015; Wong et al. 2016; Paudyal et al. 2017). There are few studies which have applied this to the post-treatment setting (King et al. 2010; Vandecaveye et al. 2012; Brenet et al 2020). Our finding of decreased absolute ADC_mean_ in patients with successful treatment differs from that observed in previous post-treatment studies (King et al. 2010) when an increased ADC_mean_ in primary tumours or lymph nodes (n = 20) was able to predict 6 month outcomes. However, King et al. only sampled visible residual tumour whilst our approach was to place standardised ROIs at the tumour location when solid tissue with increased DWI signal was not visible. This was the case for all primary tumours and the majority (32/52) of lymph nodes with definable residual disease on DWI at the relevant time points. It is speculated that the favourable outcomes with decreased absolute ADC_mean_ in our cohort actually reflects a post-treatment fibrotic response, since densely packed benign collagen may result in decreased ADC (Ailianou et al. 2018). Whilst other previous researchers have found interval changes in ADC_mean_ post treatment to have prognostic potential (Vandecaveye et al. 2012; Brenet et al. 2020) there were different methodologies and study populations.

In previous studies, ADC measurements have been performed at various intervals between 3 and 12 weeks after completion of CRT. The potential to diagnose the residual post-CRT tumour and perform salvage surgery earlier than a 12-week ^18^F-FDG PET-CT would be advantageous since surgery is less compromised by fibrosis, there is less possibility of tumour being irresectable or spreading to distant sites. This was the rationale for including a 6-week time point in our study design. The interval percentage changes in ADC_mean_ from 6 to 12-weeks were smaller than pre-treatment to 6 weeks, and the potential ability of predicting outcome with 6-weeks post-CRT absolute ADC_mean_ concurs with a previous study (King et al. 2010). It is of interest that the absolute ADC_mean_ value was predictive of 2-year and overall DFS at 6 weeks post CRT for the lymph node, whereas it was at the later 12-week time point for primary tumour. It may be speculated that there is a later differential post CRT increase in ADC_mean_ at the primary tumour site compared with lymph nodes in successfully treated patients.

HPV-OPC is increasing in incidence and now accounts for 70–80% of OPC in the United States and Western Europe (Chaturvedi et al. 2011). HPV-OPC is a clinically, epidemiologically and histologically distinct form of HNSCC; it is more radiosensitive and has a better outcome irrespective of treatment choice. It exhibits particular histopathological features such as indistinct cell borders and comedo-necrosis (El-Mofty and Lu 2003) and is characterized by an increased glucose and respiratory metabolism (Krupar et al. 2014). The potential influence of the HPV-OPC status on the prognostic values of intra or post-treatment ADC and SUV_max_ has only been addressed in a limited number of studies (Moeller et al. 2009; Ding et al. 2015; Castelli et al. 2016; Wong et al. 2016; Marzi et al. 2017; Paudyal et al. 2017; Cao et al. 2019).

The pre-treatment SUV_max_ (Kendi et al. 2015; Tahari et al. 2014) in HPV-OPC differs from that in other HNSCC. Although there are variable results (Koshkareva et al. 2014; Mowery et al. 2020), a number of studies have shown that post-treatment SUV_max_ is a less accurate predictor of outcome in HPV-OPC than other HNSCC (Moeller et al. 2009; Vainshtein et al. 2014; Castelli et al. 2016; Helsen et al. 2018). It has been speculated that the greater radio-sensitivity of HPV-OPC results in a delayed repopulation by resistant cells, and a lower sensitivity to early post-treatment detection, such that a longer interval to the surveillance ^18^F-FDG PET-CT may prove more appropriate in HPV-OPC. It has been shown that a 16-week post CRT ^18^F-FDG PET-CT demonstrates superior diagnostic accuracy for residual HPV-OPC nodal tumour when compared to 12-week ^18^F-FDG PET-CT (Liu et al. 2019). In addition, the increased cytotoxic T-cell-based immune response reported in HPV-OPC may result in spurious^18^F-FDG uptake and reduced specificity of ^18^F-FDG PET-CT.

It is also recognised that pre-treatment ADC_mean_ values are lower (Chan et al. 2016; Driessen et al. 2016) and possibly more variable in HPV-OPC (Wong et al. 2016). In our study, post-CRT ADC_mean_ interval changes were greater in HPV-OPC than other HNSCC, but the difference was only statistically significant for the primary tumour site in those with DFS. The percentage interval changes were not predictive of DFS overall or within HPV-OPC subgroups. It could therefore be argued that, without multivariate analysis to account for HPV-OPC status, the larger interval changes in treatment responders reported in previous studies (Kim et al. 2009; Berrak et al. 2011; Vandecaveye et al. 2012; King et al. 2013a, b; Matoba et al. 2014; Marzi et al. 2017; Brenet et al 2020) may be related to the predominance of prognostically favourable HPV-OPC.

Despite the small sample size, the absolute ADC_mean_ values were shown to predict 2-year and overall DFS in other HNSCC participants at both lymph node and primary tumour sites. A potential application in this group of patients is of importance since they have a poorer prognosis and will benefit most from earlier diagnosis of residual tumour. There are a few potential reasons for the failure of HPV-OPC post-CRT ADC_mean_ values to predict outcomes. First, the greater radiosensitivity of HPV-OPC and cystic nature of lymph nodes result in smaller tumour residua which are more difficult to reliably analyse. Second, there has been observed to be a wider variation in pre-treatment ADC_mean_ values in HPV-OPC (Wong et al. 2016), which may influence the ability to predict outcomes on the basis of interval change.

Previous direct comparisons of ^18^F-FDG PET-CT and quantitative DWI-MRI for their ability to predict treatment outcomes are confined to the pre-treatment and intra-treatment settings (Choi et al. 2011; Martins et al. 2015; Preda et al. 2016) or in the presence of symptomatic recurrence (Becker et al. 2018). To our best knowledge, this is the first prospective study, to date, comparing the ability of the two modalities to predict outcomes in the early post-treatment setting. Whilst a previous study showed synergy between the two modalities in stratifying the risks of therapeutic failure from pre-treatment imaging (Preda et al. 2016), this could not be reproduced in our cohort. Nonetheless, this possibility should be further explored in a larger high risk or HPV-OPC negative population.

The authors acknowledge a number of limitations in the design of this study. Firstly, the small number of both other HNSCC participants and those with treatment failure resulted in the study being sub-optimally powered for subgroup analysis. Previous publications indicated that at least 30% of HNSCC would fail treatment at loco-regional sites (Goodwin 2000) and the study was initially powered on this basis. Whilst the sample size was comparable to other similar studies, our prospectively accrued cohort comprised an unexpectedly high proportion of HPV-OPC participants (46/65) with improved outcomes. Similarly, the sample size was specifically calculated to demonstrate a differences in ADC_mean_ interval change between participants with and without 2-year DFS, so it was potentially underpowered to reveal a variation in 12-week post-CRT SUV_max_. We propose that larger cohorts are required for further validation of our results. Second, it should be noted that almost all primary tumours and the majority of lymph nodes did not demonstrate residual focal signal abnormality (> 5 mm) on DW-MRI at follow-up (4% primary tumours, 45% nodes). It has been recommended that a 5 mm lesion is required for reliable assessment of ADC in the head and neck (Theony et al. 2012). When there was no overt residual post-treatment tumour on DW-MRI, a standardised ROI was placed according to the site of the pre-treatment lesion as has been described at other tumour sites (Kuang et al. 2011). Thirdly, it was decided a priori not to correct for multiple comparisons since these were selected “planned comparisons” as part of the experimental design and not a data-driven search. It was not clear how many factors to adjust for any adjusted p value would be difficult to compute. It should, however, be appreciated that there is an inherent trade off between protecting against Type I errors and Type II errors in such an exploratory study and that a lower pre-specified significance level may not have demonstrated a predictive value of the absolute 6-week lymph node and 12-week primary tumour ADC_mean_ values. Fourthly, it is appreciated that alternative qualitative approaches using a standardised comparison with adjacent tissues may overcome the potential for false-positive ^18^ F-FDG PET results due to radiation-related inflammation. These approaches have been widely applied to the post CRT evaluation of HNSCC and studies have demonstrated their ability to predict disease outcome (Koksel et al. 2019; Krabbe et al. 2009; Marcus et al. 2014; Porceddu et al. 2011; Sj övall et al. 2016; Zhong et al. 2020). Whilst quantitative 12-week post-CRT SUV_max_ was not associated with DFS in this cohort, our results cannot be directly compared with those of qualitative interpretative ^18^ F-FDG PET criteria since they did not incorporate a comparison with the ^18^ F-FDG PET uptake in other tissues. Finally, the inter-observer agreement statistics would have been optimally obtained from the whole cohort, however, the sample analysed by two observers was noted to be representative in terms of tumour site and HPV status.

In conclusion, primary tumour and nodal absolute post-CRT ADC_mean_ measurements may predict 2-year and overall DFS in HNSCC but this does not apply to the HPV-OPC subgroup. Following successful CRT for HNSCC, percentage interval changes in ADC_mean_ at the primary tumour site are seen to differ between HPV-OPC and other HNSCC. Therefore, knowledge of HPV-OPC status is crucial to the clinical utilisation of post-CRT DWI-MRI for the prediction of outcomes.

## Supplementary Information

Below is the link to the electronic supplementary material.

Supplementary file1 (DOCX 15 kb)

## Abbreviations

ADC_mean_: Mean apparent diffusion coefficient
CRT: Chemo-radiotherapy
DFS: Disease-free survival
DWI: Diffusion-weighted imaging
HNSCC: Head and neck squamous cell cancer
HPV: Human papilloma virus
IMRT: Intensity-modulated radiotherapy
OPC: Oropharyngeal cancer
ROI: Region-of-interest
^18^F-FDG PET-CT: 18F-fluorodeoxygluocose positron emission tomography-computed tomography
SUV_max_: Maximum standardized uptake value

## References

[CR1] Agra IM, Carvalho AL, Ulbrich FS et al (2006) Prognostic factors in salvage surgery for recurrent oral and oropharyngeal cancer. Head Neck 28:107–113 16388526 10.1002/hed.20309

[CR2] Ailianou A, Mundada XP, De Perrot XT, Pusztaszieri XM, Poletti XP-A, Becker XM (2018) MRI with DWI for the detection of posttreatment head and neck squamous cell carcinoma: why morphologic MRI criteria matter. AJNR Am J Neuroradiol 39:748–755 29449279 10.3174/ajnr.A5548 PMC7410767

[CR3] Becker M, Varoquaux AD, Combescure C et al (2018) Local recurrence of squamous cell carcinoma of the head and neck after radio(chemo)therapy: diagnostic performance of FDG-PET/MRI with diffusion-weighted sequences. Eur Radiol 28:651–663 28812148 10.1007/s00330-017-4999-1 PMC5740208

[CR4] Berrak S, Chawla S, Kim S et al (2011) Diffusion weighted imaging in predicting progression free survival in patients with squamous cell carcinomas of the head and neck treated with induction chemotherapy. Acad Radiol 18:1225–1232 21835649 10.1016/j.acra.2011.06.009 PMC3168957

[CR5] Brenet E, Barbe C, Hoeffel C et al (2020) Predictive value of early post-treatment diffusion-weighted MRI for recurrence or tumor progression of head and neck squamous cell carcinoma treated with chemo-radiotherapy. Cancer 12:1234 10.3390/cancers12051234 PMC7281260 32422975

[CR6] Cao Y, Aryal M, Li P et al (2019) Predictive values of MRI and PET derived quantitative parameters for patterns of failure in both p16+ and p16- high risk head and neck cancer. Front Oncol 14:1118 10.3389/fonc.2019.01118 PMC6874128 31799173

[CR7] Castelli J, De Bari B, Depeursinge A et al (2016) Overview of the predictive value of quantitative 18 FDG PET in head and neck cancer treated with chemoradiotherapy. Crit Rev Oncol Hematol 108:40–51 27931839 10.1016/j.critrevonc.2016.10.009

[CR8] Chan JYK, Sanguineti G, Richmon JD, Marur S (2012) Retrospective review of positron emission tomography with contrast-enhanced computed tomography in the posttreatment setting in human papillomavirus–associated oropharyngeal carcinoma. Arch Otolaryngol Head Neck Surg 138:1040–1046 23165378 10.1001/jamaoto.2013.607 PMC3715070

[CR9] Chan MW, Higgins K, Enepekides D et al (2016) Radiologic differences between human papillomavirus-related and human papillomavirus-unrelated oropharyngeal carcinoma on diffusion-weighted imaging. ORL J Otorhinolaryngol Relat Spec 78:344–352 28245461 10.1159/000458446

[CR10] Chang JH, Wu CC, Yuan KSP, Wu ATH, Wu SY (2017) Loco-regionally recurrent head and neck squamous cell carcinoma: incidence, survival, prognostic factors and treatment outcomes. Oncotarget 8:55600–55612 28903447 10.18632/oncotarget.16340 PMC5589686

[CR11] Chaturvedi AK, Engels EA, Pfeiffer RM et al (2011) Human papillomavirus and rising oropharyngeal cancer incidence in the United States. J Clin Oncol 29:4294–4301 21969503 10.1200/JCO.2011.36.4596 PMC3221528

[CR12] Chawla S, Kim S, Wang S, Poptani H (2009) Diffusion weighted imaging in head and neck cancers. Future Oncol 5:959–975 19792966 10.2217/fon.09.77 PMC2791671

[CR13] Chernock RD, El-Mofty SK, Thorstad WL, Parvin CA, Lewis JS (2009) HPV-related nonkeratinizing squamous cell carcinoma of the oropharynx: utility of microscopic features in predicting patient outcome. Head Neck Pathol 3:186–194 20596971 10.1007/s12105-009-0126-1 PMC2811624

[CR14] Choi SH, Paeng JC, Sohn CH et al (2011) Correlation of 18F-FDG uptake with apparent diffusion coefficient ratio measured on standard and high b value diffusion MRI in head and neck cancer. J Nucl Med 52:1056–1062 21680692 10.2967/jnumed.111.089334

[CR15] Ding Y, Hazle JD, Mohamed ASR et al (2015) Intravoxel incoherent motion imaging kinetics during chemoradiotherapy for human papillomavirus-associated squamous cell carcinoma of the oropharynx: preliminary results form a prospective pilot study. NMR Biomed 28:1645–1654 26451969 10.1002/nbm.3412 PMC4715635

[CR16] Driessen JP, van Bemmel AJ, van Kempen PM et al (2016) Correlation of human papillomavirus status with apparent diffusion coefficient of diffusion-weighted MRI in head and neck squamous cell carcinomas. Head Neck 38:E613-618 25783872 10.1002/hed.24051

[CR17] El-Mofty SK, Lu DW (2003) Prevalence of human papillomavirus type 16 DNA in squamous cell carcinoma of the palatine tonsil, and not the oral cavity, in young patients: a distinct clinicopathologic and molecular disease entity. Am J Surg Pathol 27:1463–1470 14576481 10.1097/00000478-200311000-00010

[CR18] Fakhry C, Lacchetti C, Rooper LM et al (2018) Papillomavirus testing in head and neck carcinomas: ASCO Clinical Practice Guideline Endorsement of the College of American Pathologists Guideline. J Clin Oncol 36:3152–3161 30188786 10.1200/JCO.18.00684

[CR19] Ferlay J, Shin HR, Bray F, Forman D, Mathers C, Parkin DM (2010) Estimates of worldwide burden of cancer in 2008: GLOBOCAN 2008. Int J Cancer 127:2893–2917 21351269 10.1002/ijc.25516

[CR20] Galbán CJ, Lemasson L, Hoff BA et al (2015) Development of a multiparametric voxel-based magnetic resonance imaging biomarker for early cancer therapeutic response assessment. Tomography 1:44–52 26568982 10.18383/j.tom.2015.00124 PMC4643274

[CR21] Goodwin WJ Jr (2000) Salvage surgery for patients with recurrent squamous cell carcinoma of the upper aerodigestive tract: when do the ends justify the means? Laryngoscope 110:1–18 10714711 10.1097/00005537-200003001-00001

[CR22] Helsen N, Van den Wyngaert T, Carp L, Stroobants S (2018) FDG-PET/CT for treatment response assessment in head and neck squamous cell carcinoma: a systematic review and meta-analysis of diagnostic performance. EJNMMI 45:1063–1071 10.1007/s00259-018-3978-3 29478080

[CR23] Hermans R, Pameijer FA, Mancuso AA, Parsons JT, Mendenhall WM (2000) Laryngeal or hypopharyngeal squamous cell carcinoma: can follow-up CT after definitive radiation therapy be used to detect local failure earlier than clinical examination alone? Radiology 214:683–687 10715030 10.1148/radiology.214.3.r00fe13683

[CR24] Kendi AT, Magliocca K, Corey A et al (2015) Do 18F-FDG PET/CT parameters in oropharyngeal and oral cavity squamous cell carcinomas indicate HPV status? Clin Nucl Med 40:e196–e200 25608156 10.1097/RLU.0000000000000691 PMC4360134

[CR25] Kim S, Loevner L, Quon H et al (2009) Diffusion-weighted magnetic resonance imaging for predicting and detecting early response to chemoradiation therapy of squamous cell carcinomas of the head and neck. Clin Cancer Res 15:986–994 19188170 10.1158/1078-0432.CCR-08-1287 PMC2673914

[CR26] Kim R, Ock CY, Keam B et al (2016) Predictive and prognostic value of PET/CT imaging post-chemoradiotherapy and clinical decision-making consequences in locally advanced head & neck squamous cell carcinoma: a retrospective study. BMC Cancer 16:116 26884055 10.1186/s12885-016-2147-y PMC4756525

[CR27] King AD, Mo FK, Yu KH et al (2010) Squamous cell carcinoma of the head and neck: diffusion-weighted MR imaging for prediction and monitoring of treatment response. Eur Radiol 20:2213–2220 20309553 10.1007/s00330-010-1769-8

[CR28] King AD, Chow K-K, Yu K-H et al (2013a) Head and neck squamous cell carcinoma: diagnostic performance of diffusion-weighted MR imaging for the prediction of treatment response. Radiology 266:531–538 23151830 10.1148/radiol.12120167

[CR29] King AD, Keung CK, Yu KH et al (2013b) T2-weighted MR imaging early after chemoradiotherapy to evaluate treatment response in head and neck squamous cell carcinoma. AJNR Am J Neuroradiol 34:1237–1241 23306012 10.3174/ajnr.A3378 PMC7964595

[CR30] Koksel Y, Gencturk M, Spano A et al (2019) Utility of Likert scale (Deauville criteria) in assessment of chemoradiotherapy response of primary oropharyngeal squamous cell cancer site. Clin Imaging 55:89–94 30771643 10.1016/j.clinimag.2019.01.007

[CR31] Koshkareva Y, Branstetter BB, Gaughan JP, Ferris RL (2014) Predictive accuracy of first post-treatment PET/CT in HPV-related oropharyngeal squamous cell carcinoma. Laryngoscope 124:1843–1847 24474646 10.1002/lary.24617

[CR32] Krabbe CA, Prium J, Dijkstra PU et al (2009) 18F-FDG PET as a routine posttreatment surveillance tool in oral and oropharyngeal squamous cell carcinoma: a prospective study. J Nucl Med 50:1940–1947 19910434 10.2967/jnumed.109.065300

[CR33] Krupar R, Robold K, Gaag D et al (2014) Immunologic and metabolic characteristics of HPV-negative and HPV-positive head and neck squamous cell carcinomas are strikingly different. Virchows Arch 465:299–312 25027580 10.1007/s00428-014-1630-6

[CR34] Kuang F, Yan Z, Wang J, Rao Z (2011) The value of diffusion-weighted MRI to evaluate the response to radiochemotherapy for cervical cancer. Magn Reson Imaging 32:342–349 10.1016/j.mri.2013.12.007 24512795

[CR35] Liu Y-H, Milne R, Lock G, Panizza B (2019) Utility of a repeat PET/CT scan in HPV-associated oropharyngeal cancer following incomplete nodal response from (chemo)radiotherapy. Oral Oncol 88:153–159 30616786 10.1016/j.oraloncology.2018.11.033

[CR36] Marcus C, Ciarollo A, Tahari AK et al (2014) Head and neck PET/CT: therapy response interpretation criteria (Hopkins Criteria)–interreader reliability, accuracy, and survival outcomes. J Nucl Med 55:1411–1416 24947059 10.2967/jnumed.113.136796 PMC4390037

[CR37] Martins BL, Chojniak R, Kowalski LP, Nicolau UR, Lima ENP, Bitencourt AGV (2015) Diffusion-weighted MRI in the assessment of early treatment response in patients with squamous-cell carcinoma of the head and neck: comparison with morphological and PET/CT findings. PLoS ONE 10:e0140009 26562784 10.1371/journal.pone.0140009 PMC4643009

[CR38] Marzi S, Piludu F, Sanguineti G et al (2017) The prediction of the treatment response of cervical nodes using intravoxel incoherent motion diffusion-weighted imaging. Eur J Radiol 92:93–102 28624026 10.1016/j.ejrad.2017.05.002

[CR39] Matoba M, Tuji H, Shimode Y et al (2014) Fractional change in apparent diffusion coefficient as an imaging biomarker for predicting treatment response in head and neck cancer treated with chemoradiotherapy. AJNR Am J Neuroradiol 35:379–385 24029391 10.3174/ajnr.A3706 PMC7965773

[CR40] Matoba M, Tuji H, Shimode Y, Kondo T, Oota K, Tonami H (2017) The role of changes in maximum standardized uptake value of FDG PET-CT for posttreatment surveillance in patients with head and neck squamous cell carcinoma treated with chemoradiotherapy: preliminary findings. Br J Radiol 90:20150404 28055245 10.1259/bjr.20150404 PMC5601519

[CR41] Mehanna H, Wong WL, McConkey C et al (2016) PET-CT surveillance versus neck dissection in advanced head and neck cancer. N Engl J Med 374:1444–1454 27007578 10.1056/NEJMoa1514493

[CR42] Moeller BJ, Rana V, Cannon BA et al (2009) Prospective risk-adjusted [18F] fluorodeoxyglucose positron emission tomography and computed tomography assessment of radiation response in head and neck cancer. J Clin Oncol 27:2509–2515 19332725 10.1200/JCO.2008.19.3300 PMC2739610

[CR43] Mowery YM, Vergalasova I, Rushing CN et al (2020) Early ^18^F-FDG-PET response during radiation therapy for HPV-related oropharyngeal cancer may predict disease recurrence. Int J Rad Oncol Biol Phys 108:969–976 10.1016/j.ijrobp.2020.08.029 32800802

[CR44] Paudyal R, Hun J, Riaz N et al (2017) Intravoxel incoherent motion diffusion-weighted MRI during chemoradiation therapy to characterize and monitor treatment response in human papillomavirus head and neck squamous cell carcinoma. JMRI 45:1013–1023 27862553 10.1002/jmri.25523 PMC5363344

[CR45] Porceddu SV, Pryor DI, Burmeister E et al (2011) Results of a prospective study of positron emission tomography-directed management of residual nodal abnormalities in node-positive head and neck cancer after definitive radiotherapy with or without systemic therapy. Head Neck 33:1675–1682 22076976 10.1002/hed.21655

[CR46] Preda L, Conte G, Bonello L et al (2016) Combining standardized uptake value of FDG-PET and apparent diffusion coefficient of DW-MRI improves risk stratification in head and neck squamous cell carcinoma. Eur Radiol 26:4432–4441 26965504 10.1007/s00330-016-4284-8

[CR47] Schouten CS, de Bree R, van der Putten L et al (2014) Diffusion-weighted EPI- and HASTE-MRI and 18F-FDG-PET-CT early during chemoradiotherapy in advanced head and neck cancer. Quant Imaging Med Surg 4:239–250 25202659 10.3978/j.issn.2223-4292.2014.07.15 PMC4137183

[CR48] Sheikhbahaei S, Taghipour M, Ahmad R et al (2015) Diagnostic accuracy of follow-up FDG PET or PET/CT in patients with head and neck cancer after definitive treatment: A systematic review and meta-analysis. Am J Roentgenol 205:629–639 26295652 10.2214/AJR.14.14166

[CR49] Sherriff JM, Ogunremi B, Colley S, Sanghera P, Hartley A (2012) The role of positron emission tomography/CT imaging in head and neck cancer patients after radical chemoradiotherapy. Br J Radiol 85:e1120–e1126 22744325 10.1259/bjr/20976707 PMC3500811

[CR50] Sjövall J, Bitzen U, Kjellen E et al (2016) Qualitative interpretation of PET scans using a Likert scale to assess neck node response to radiotherapy in head and neck cancer. Eur j Nucl Med Mol Imaging 43:609–616 26428529 10.1007/s00259-015-3194-3

[CR51] Tahari AK, Alluri KC, Quon H, Koch W, Wahl RL, Subramaniam RM (2014) FDG PET/CT imaging of oropharyngeal squamous cell carcinoma: characteristics of human papillomavirus-positive and -negative tumors. Clin Nucl Med 39:225–231 24152652 10.1097/RLU.0000000000000255 PMC4074504

[CR52] Theony HC, De Keyzer F, King AD (2012) Diffusion-weighted MR imaging in the head and neck. Radiology 263:19–32 22438440 10.1148/radiol.11101821

[CR53] Vainshtein JM, Spector ME, Stenmark MH et al (2014) Reliability of post-chemoradiotherapy F-18-FDG PET/CT for prediction of locoregional failure in human papillomavirus-associated oropharyngeal cancer. Oral Oncol 50:234–239 24387978 10.1016/j.oraloncology.2013.12.003 PMC4159357

[CR54] Vandecaveye V, Dirix P, De Keyzer F et al (2012) Diffusion-weighted magnetic resonance imaging early after chemoradiotherapy to monitor treatment response in head-and-neck squamous cell carcinoma. Int J Radiat Oncol Biol Phys 82:1098–1107 21514067 10.1016/j.ijrobp.2011.02.044

[CR55] Wong KH, Panek R, Welsh L et al (2016) The predictive value of early assessment after 1 cycle of induction chemotherapy with 18F-FDG PET/CT and diffusion-weighted MRI for response to radical chemoradiotherapy in head and neck squamous cell carcinoma. J Nucl Med 57:1843–1850 27417648 10.2967/jnumed.116.174433

[CR56] Zhang I, Branstetter BF, Beswick DM et al (2010) The Benefit of early PET/CT surveillance in HPV-associated head and neck squamous cell carcinoma. Arch Otolaryngol Head Neck Surg 137:1106–1111 10.1001/archoto.2011.181 22106234

[CR57] Zhang Q, Zhou Z, Qin G et al (2018) Prediction of local persistence/recurrence on PET/CT scans after radiation therapy treatmnet of head and neck cancer using a multi-objective radiomics model. Int J Rad Oncol Bio Phys 102:S94

[CR58] Zhong J, Sundersingh M, Dyker K et al (2020) Post-treatment FDG PET-CT in head and neck carcinoma: comparative analysis of 4 qualitative interpretative criteria in a large patient cohort. Sci Rep 10:4086 32139722 10.1038/s41598-020-60739-3 PMC7058010

