# Supplementary material for: The ability of post-chemoradiotherapy DWI ADCmean and 18F-FDG SUVmax to predict treatment outcomes in head and neck cancer: impact of human papilloma virus oropharyngeal cancer status
Source: J Cancer Res Clin Oncol. 2021 Jun 22;147(8):2323–36. doi: 10.1007/s00432-021-03662-y (PMC8236463; doi:10.1007/s00432-021-03662-y)
Supplement: Supplementary file 1 — Supplementary file1 (DOCX 15 kb) [file 432_2021_3662_MOESM1_ESM.docx]

Supplementary table

MRI protocol and ^18^F-FDG PET/CT protocols

| MRI protocol | | | | | | | | | |
| --- | --- | --- | --- | --- | --- | --- | --- | --- | --- |
| Sequence | Plane | Slice thickness/ gap | TR/TE | Field of view | Number of averages | Pixel Bandwidth | Flip angle | | Acquisition matrix |
| T1w | axial | 4/0 | 549/11 | 220x220 | 1 | 200 | 160 | | 384/269 |
| T2w | axial | 4/0 | 5830/102 | 220x220 | 1 | 190 | 150 | | 384/346 |
| T1w fat saturated -DIXON  post gadolinium | axial | 4/0 | 566/11 | 220x220 | 1 | 330 | 145 | | 320x224 |
| STIR | coronal | 3/0.3 | 3000/35  TI 140 | 260X260 | 1 | 220 | 160 | | 320X224 |
| T1w fat sat-DIXON  post gadolinium | coronal | 3/0.3 | 708/10 | 280X280 | 1 | 340 | 145 | | 320X320 |
| Diffusion weighted imaging | Axial (two slabs) | 4/0.5 | 5900/60  b values 0,50,100,800,1500 s/mm ^2^ | 240x240 | 2 | 1375 | 90 | | 130 x130 |
| ^18^F-FDG PET/CT | | | | | | | | | |
| PET/CT scanner | Algorithm | | Post-filter | Matrix size | | Pixel Spacing | | Low dose CT scan | |
| Siemens mCT Flow VST | OSEM, time-of-flight, 2 iterations 21 subsets | | Gaussian 6.4mm FWHM | 200 x 200 x 46 | | 3.07 x 4.07 x 2 | | 140 kV, 10 mA, 0.5 s rotation time, 40 mm collimation | |
| GE Discovery DST 710 | ‘VPFX’, OSEM, time-of-flight, 2 iterations 24 subsets | | Gaussian 5.0mm FWHM | 256 x 256 x 47 | | 2.73 x 2.73 x 3.27 | | 140 kV, 10 mA, 0.5 s rotation time, 40 mm collimation | |

OSEM: Ordered subset expectation maximisation

TR: Repetition time

TE: Echo time

FWHM: Full width half maximum

kV kilovoltage

mA milliampere
